# A 7-Year Analysis of the U.S. FDA Good Clinical Practice Inspection Outcomes for Marketing Applications

**DOI:** 10.1007/s43441-025-00835-6

**Published:** 2025-07-19

**Authors:** Courtney McGuire, Jenn W. Sellers, Laurie Muldowney

**Affiliations:** Good Clinical Practice Assessment Branch (GCPAB), Office of Scientific Investigations (OSI) Office of Compliance (OC), Center for Drug Evaluation and Research (CDER), U.S. Food and Drug Administration, 10903 New Hampshire Ave, Silver Spring, MD 20993 USA

## Abstract

**Background:**

The U.S. Food and Drug Administration (FDA) conducts Good Clinical Practice (GCP) inspections to support regulatory decisions on marketing applications. Inspection outcomes including issuance of Form FDA 483 and a comparison of recommended and final inspection classifications have never been reported.

**Methods:**

GCP inspections conducted from calendar year 2017 to 2023 were analyzed by characteristics and outcomes. The recommended and final inspection classifications were compared to determine no change, upgrades, or downgrades. The inspection deficiency areas for upgrades and downgrades were summarized.

**Results:**

The FDA completed 2836 review-based routine GCP inspections in support of Center for Drug Evaluation and Research (CDER) marketing applications between 2017 and 2023. Overall, final no action indicated (NAI), voluntary action indicated (VAI), and official action indicated (OAI) classifications were determined for 81.2%, 18.5%, and 0.3% of all inspections (The majority of final OAI classifications are driven by for-cause GCP inspections rather than review-based GCP inspections). The percent of establishments issued a Form FDA 483 decreased from 2017 (23.5%) to 2023 (10.4%). Roughly 97% of recommended NAI and 96% of recommended VAI classifications received the same final classification, but there was an increase of VAI to NAI downgrades during years 2020 to 2022. Common deficiency areas related to both upgrades and downgrades were adherence to trial protocol and adequacy of records.

**Conclusions:**

Nearly all (99.7%) inspections were classified as either NAI or VAI during the study period, and their recommended and final classifications remained highly congruent. The Coronavirus Disease 19 (COVID-19) pandemic likely impacted the number of VAI to NAI downgrades.

## Introduction

The U.S. Food and Drug Administration’s (FDA) use and analysis of data from clinical trials submitted in support of marketing applications are dependent on the knowledge that the data are reliable for regulatory decision making. Good clinical practice (GCP) inspections evaluate the conduct of clinical trials to ensure that the protocols and related plans are followed and that regulated entities comply with the FDA laws and regulations governing human subject protection and the conduct of clinical trials [1–4]. GCP inspections of Sponsors, Contract Research Organizations (CRO), Clinical Investigators (CI), and Sponsor-Investigators (SI) are conducted under FDA’s Bioresearch Monitoring (BIMO) Program. GCP inspections conducted in support of a marketing application, also referred to as routine and review-based inspections, are normally preannounced and focus on the conduct of specific clinical trial(s) submitted to Center for Drug Evaluation and Research (CDER) as part of a marketing application. In contrast, for-cause inspections, which are conducted to evaluate potential noncompliance or safety issues raised in a complaint, are normally not preannounced and may assess the conduct of multiple trials conducted at an establishment [5, 6].

The Office of Scientific Investigations (OSI) within CDER and the Office of Inspections and Investigations (OII, formerly Office of Regulatory Affairs) work together to oversee regulated establishments. Pivotal clinical trials included in a marketing application may be conducted at multiple trial sites around the world, and typically only a few establishments are selected for inspections. OSI issues GCP inspection assignments using a risk-based approach to determine whether and which establishments to inspect to support the review of marketing applications. OII investigators conduct review-based inspections following the relevant compliance program [2, 3] and focus on specific areas identified by OSI in the inspection assignment memo. During the inspections, OII investigators may observe objectionable conditions they deem to be FDA regulatory violations. These observations are listed and described on an FDA Form 483 (Inspectional Observations, 483), which is issued to the establishment at the closing of the inspection. Following the inspection, the OII investigator writes the Establishment Inspection Report (EIR) and makes one of the following classification recommendations to OSI: (1) no action indicated (NAI), i.e., no objectionable conditions or practices were found during the inspection; (2) voluntary action indicated (VAI), i.e., objectionable conditions or practices were found, but the FDA is not prepared to take or recommend any administrative or regulatory action; (3) official action indicated (OAI), i.e., regulatory and/or administrative actions are recommended. OII usually recommends VAI or OAI when a Form FDA 483 is issued.

OSI reviews the information provided by OII, including the EIR, exhibits, and if applicable, the Form FDA 483 and inspected establishment’s response, to determine the final classification for the inspection. Final classification determinations for GCP inspections require close assessment of the specific language in the protocol when determining whether observations on inspections were regulatory violations. Final classification determinations also consider the impact of the violation on participant rights and safety and the reliability and interpretation of critical trial data or processes. This determination requires a detailed understanding of the marketing application, including but not limited to the investigational product, indication being studied, trial protocol, and trial results. For example, the impact of an unreported adverse event may be different in a trial of a novel unapproved product compared to a trial of an already approved product with a well-established safety profile. The determination of the significance of any observed regulatory violations by OSI is a critical component of the compliance review of GCP inspection findings and may result in changes from the recommended classification to final classification. OSI also provides recommendations to the CDER Review Divisions on the reliability of clinical trial data in marketing applications in a Clinical Inspection Summary (CIS) based on the inspection findings.

In this paper, we report the outcomes from review-based routine GCP inspections including issuance of Form FDA 483s, recommended and final classifications, upgrades and downgrades, and related deficiencies. 

## Methods

This was a retrospective descriptive study of review-based routine GCP inspection outcomes of CI, SI, Sponsor, and CRO inspections conducted in support of CDER New Drug Applications (NDAs), Biologics License Applications (BLAs), and Emergency Use Authorizations (EUAs)^1^ between calendar years (CY) 2017 and 2023, inclusive. In 2017, the FDA initiated the Office of Regulatory Affairs realignment, transitioning from a geographically based structure to a program-based management model. This restructuring established a specialized office which focuses on GCP inspections [7–9]. As a result, 2017 was selected as the starting year for this study. The data sources included the following FDA internal databases: the Document Archiving, Reporting & Regulatory Tracking System (DARRTS), Compliance Program Information System (COMPLIS), Enterprise Content Management System (ECMS), and Online Search and Retrieval System (OSAR). Data analyses were conducted in JMP 17 and Microsoft Excel for Microsoft 365 MSO (Version 2302).

GCP inspection data were identified by data queries in COMPLIS based on the inspection start date between CY 2017–2023. The following extracted data were summarized with descriptive statistics: application type (NDA, BLA, or IND), review designation (Priority or Standard), submission type (original or supplement), location (domestic or foreign), establishment type (CI, SI, Sponsor, or CRO), issuance of Form FDA 483 (yes or no), recommended classification (NAI, VAI, or OAI), and final classification (NAI, VAI, or OAI).

For each inspection, the recommended and final inspection classifications were compared, and the results were categorized as unchanged, downgrade, or upgrade. Unchanged was defined as identical recommended and final inspection classifications. Downgrade was defined as a change from a recommended classification of OAI to a final classification of VAI or NAI or from a recommended classification of VAI to a final classification of NAI. Upgrade was defined as a change from a recommended classification of NAI to a final classification of VAI or OAI or from a recommended classification of VAI to a final classification of OAI.

Inspection findings were grouped into deficiency areas to better characterize the types of observations made on inspections. For upgrades, the CIS and the FDA’s correspondences to the establishments were reviewed to extract information on the final inspection deficiency(s). For downgrades, the Form FDA 483 and EIRs were reviewed to extract information on the observed inspection deficiency(s). The individual inspection deficiencies were grouped by general deficiency areas (i.e., adherence to trial protocol, adequacy of records, drug accountability, reporting of safety information, monitoring / trial oversight, Institutional Review Board [IRB] approval, informed consent, or other), and the distribution of the deficiency areas were separately summarized for upgrades and downgrades, counting each deficiency area once per upgraded or downgraded inspection.

Inspections excluded from the analyses included those inspections canceled or changed to investigations, those with duplicates or incomplete information, or refusal on the part of the establishment. Inspections conducted in response to a complaint or required report (i.e., for cause inspections) and inspections of bioavailability/bioequivalence studies were also excluded. Finally, remote examinations of establishments (i.e., remote regulatory assessments [RRA]) were excluded because RRAs are not considered inspections under Sect. 704 of the Federal Food, Drug, and Cosmetic (FD&C) Act [10], and the outcomes of RRAs were not classified during the study period. A description of the use of RRAs to evaluate GCP during the Coronavirus Disease 19 (COVID-19) public health emergency was previously published [11].

## Results

### Data Collected for Analyses

**Fig. 1:**
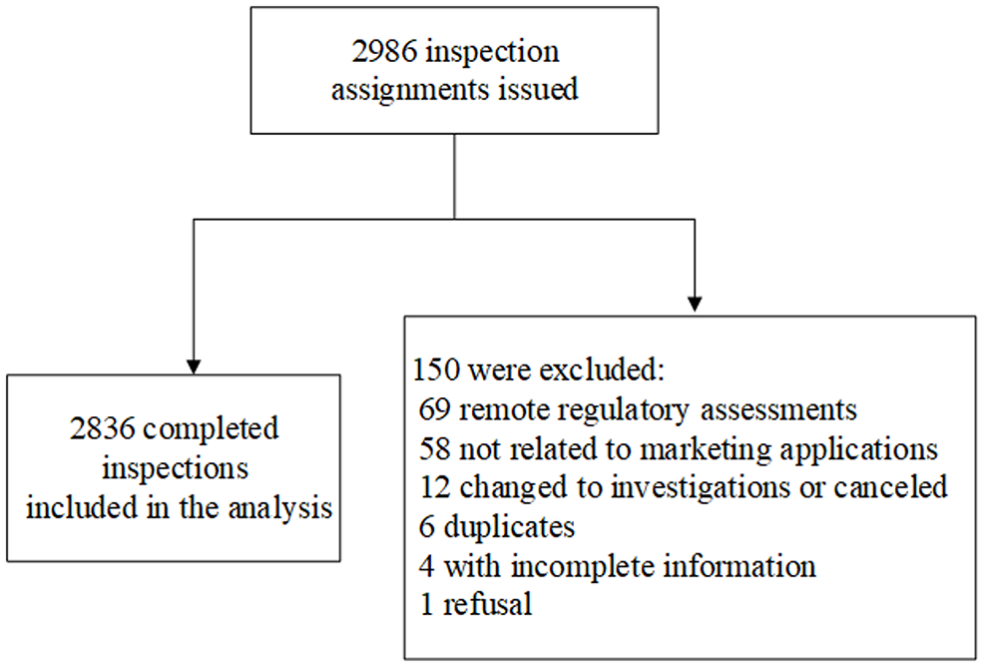
CDER Reviewed-Based Routine GCP Inspection Data Summary

A total of 2986 GCP inspection assignments were identified with an inspection start date between 2017 and 2023, inclusive. One hundred fifty inspections were excluded for the reasons listed in Fig. 1. A total of 2836 completed inspections of CIs, SIs, Sponsors, and CROs conducted in support of NDAs, BLAs, and EUAs submitted to CDER were included and reviewed for the data analyses (Fig. 1).

### Characteristics of GCP Inspections for 2017 to 2023

Of the 2836 total review-based routine GCP inspections included in the analysis, 71.0% were conducted in support of NDAs, 28.6% BLAs, and 0.4% EUAs (submitted under the IND). The majority of GCP inspections were for original applications (73.6%), and overall, the distribution of priority and standard review designation was approximately equal. Most inspections were of CIs (85.9%) and were conducted in the U.S. (70.8%) (Table 1).

**Table 1:** Summary for CDER Review-based GCP Inspections Included in the Analysis, 2017 to 2023

**a. Application Characteristics **								
		**Application Type**			**Review Designation**		**Submission Type**	
		NDA	BLA	IND^1^	Priority	Standard	Original	Supplement
n		2013	812	11	1460	1376	2086	750
%^2^		71.0%	28.6%	0.4%	51.5%	48.5%	73.6%	26.4%
**b. Inspection Characteristics**								
	**Inspection Location**				**Establishment Type**			
	Domestic			Foreign	CI	Sponsor	CRO	SI
n	2009			827	2435	275	122	4
%^2^	70.8%			29.2%	85.9%	9.7%	4.3%	0.1%

n = number of inspections in each category; N = the total number of inspections, 2836. ^1^Includes EUAs related to public health emergency submitted and reviewed under the associated IND. ^2^Percentage calculated as n/N for each category

**Fig. 2:**
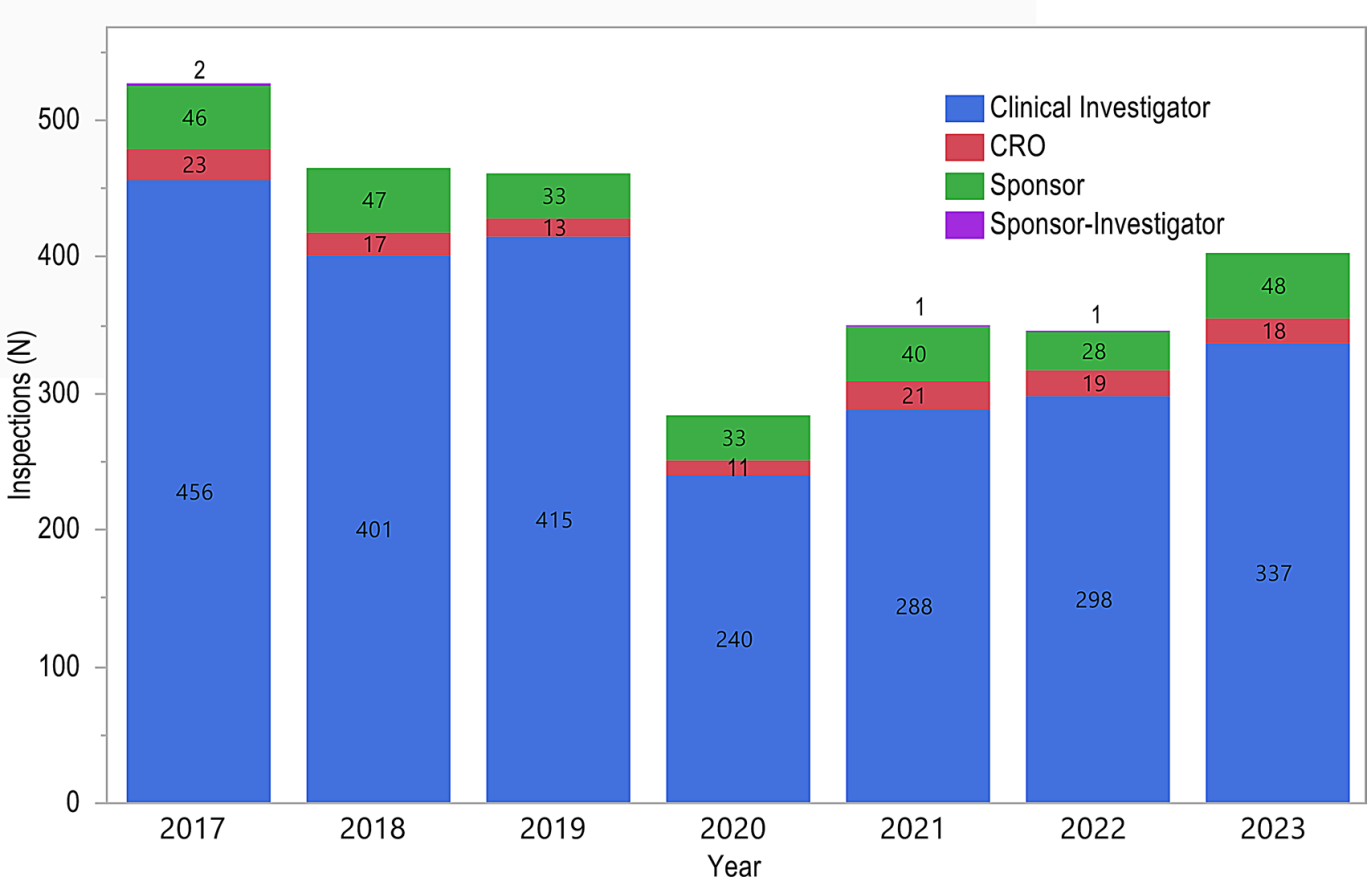
Inspection Establishment Type by Year

The total number of inspections completed per year varied over the 7-year time period, with the maximum occurring in 2017 (527 inspections) and the minimum in 2020 (284 inspections). The distribution of inspections by establishment type remained relatively consistent over the 7-year period; each year, the majority of GCP inspections were for CIs (82.3 to 90.0%) followed by Sponsors (7.2 to 11.9%), CROs (2.8 to 6.0%), and SIs (0 to 0.4%) (Fig. 2).

**Fig. 3:**
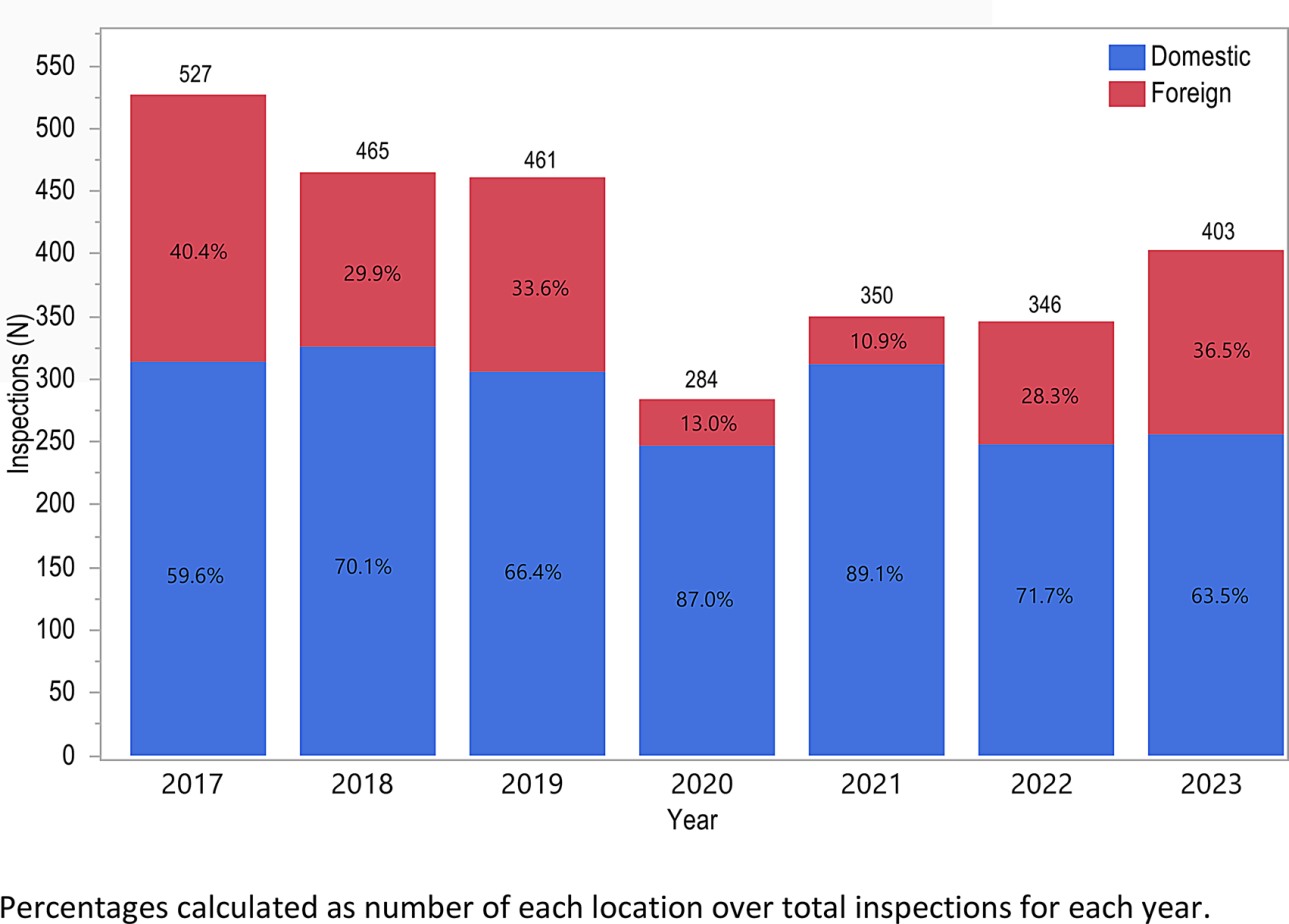
Inspection Location by Year

The number of foreign inspections followed the distribution of total inspections completed per year, also reaching a nadir in 2020 (37 foreign inspections), but the number of domestic inspections remained relatively consistent (Fig. 3).

**Fig. 4:**
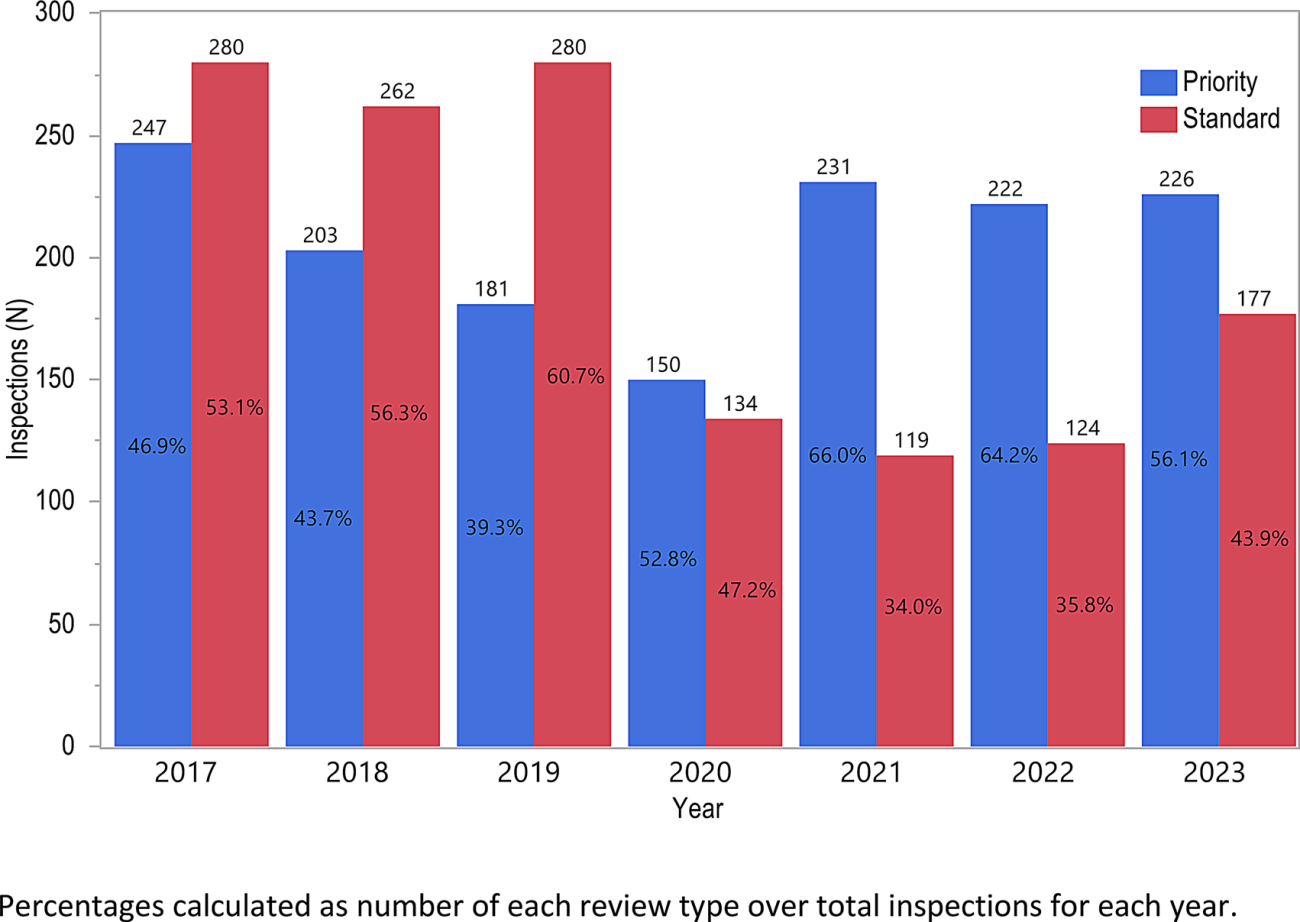
Marketing Application Review Type by Year

Inspections for standard reviews exceeded priority reviews between 2017 and 2019, but the opposite distribution occurred between 2020 and 2023 (Fig. 4).

### Summary of Form FDA 483s Issued by Year

A Form FDA 483 was issued for 17.5% of GCP inspections completed during the 7-year time period. By year, the highest percent of inspections with a Form FDA 483 issued occurred in 2017 (23.5%). This percentage generally decreased over time, with the lowest percent issued in 2022 (12.4%) and 2023 (10.4%) (Table 2).

**Table 2:** Number and Percentage (%) of Form FDA 483 Issued by Year

Year	Form FDA 483 Issued / Total Inspections (%)
All	496/2836 (17.5%)
2017	124/527 (23.5%)
2018	89/465 (19.1%)
2019	90/461 (19.5%)
2020	44/284 (15.5%)
2021	64/350 (18.3%)
2022	43/346 (12.4%)
2023	42/403 (10.4%)

### Summary of Recommended and Final Inspection Classifications

During the 7-year time period, a classification of NAI, VAI, and OAI was recommended for 82.5%, 16.6%, and 0.9% of all GCP inspections, respectively. Recommended NAI classifications represented the highest percent of inspections every year and showed an upward trend from 2017 (76.3%) to 2023 (89.6%). In contrast, the highest percent of recommended VAI classifications was in 2017 (23.0%) and the lowest in 2023 (9.9%). Recommended OAI classifications remained infrequent during the 7-year period.

A majority of inspections received a final classification of NAI (81.2%) during the 7-year time period, with a final classification of VAI representing the second largest category (18.5%). OAI final inspection classifications occurred infrequently and at a similar incidence each year, representing less than 1% of all inspections during the 7-year time period. The distribution of final NAI and VAI classifications varied over the time period, with the overall percent of NAI trending up (74.8–87.8%) and VAI trending down (25.2–11.7%) from 2017 to 2023, respectively (Table 3).

**Table 3:** Recommended and Final Classification of Inspections by Year, Location, and Establishment Type

**a. Year**								
		**Recommended Classification**				**Final Classification**		
Year	N	NAI	VAI		OAI	NAI	VAI	OAI
All	2836	2339 (82.5%)	471 (16.6%)		26 (0.9%)	2302 (81.2%)	525 (18.5%)	9 (0.3%)
2017	527	402 (76.3%)	121 (23.0%)		4 (0.8%)	394 (74.8%)	133 (25.2%)	0
2018	465	376 (80.9%)	82 (17.6%)		7 (1.5%)	369 (79.4%)	96 (20.6%)	0
2019	461	371 (80.5%)	86 (18.6%)		4 (0.9%)	361 (78.3%)	99 (21.5%)	1 (0.2%)
2020	284	240 (84.5%)	41 (14.4%)		3 (1.1%)	241 (84.9%)	41 (14.4%)	2 (0.7%)
2021	350	286 (81.7%)	60 (17.1%)		4 (1.1%)	282 (80.6%)	66 (18.9%)	2 (0.6%)
2022	346	303 (87.6%)	41 (11.8%)		2 (0.6%)	301 (87.0%)	43 (12.4%)	2 (0.6%)
2023	403	361 (89.6%)	40 (9.9%)		2 (0.5%)	354 (87.8%)	47 (11.7%)	2 (0.5%)
Pre-COVID^1^	1486	1177 (79.2%)	294 (19.8%)		15 (1.0%)	1152 (77.5%)	333 (22.4%)	1 (0.1%)
COVID^2^	1114	942 (84.6%)	163 (14.6%)		9 (0.8%)	931 (83.6%)	177 (15.9%)	6 (0.5%)
**b. Location**								
		**Recommended Classification**				**Final Classification**		
Location	N	NAI	VAI	OAI		NAI	VAI	OAI
Domestic	2009	1630 (81.1%)	363 (18.1%)	16 (0.8%)		1603 (79.8%)	401 (19.9%)	5 (0.2%)
Foreign	827	709 (85.7%)	108 (13.1%)		10 (1.2%)	699 (84.5%)	124 (15.0%)	4 (0.5%)
**c. Establishment Type (ET)**								
		**Recommended Classification**				**Final Classification**		
ET	N	NAI	VAI		OAI	NAI	VAI	OAI
CI	2435	2000 (82.1%)	414 (17.0%)		21 (0.9%)	1973 (81.0%)	454 (18.6%)	8 (0.3%)
Sponsor	275	221 (80.4%)	50 (18.2%)		4 (1.5%)	213 (77.4%)	61 (22.2%)	1 (0.4%)
CRO	122	115 (94.3%)	6 (4.9%)		1 (0.8%)	112 (91.8%)	10 (8.2%)	0
SI	4	3 (75.0%)	1 (25.0%)		0	4 (100.0%)	0	0

CI = Clinical Investigator; CRO = Contract Research Organization; SI = Sponsor-Investigator. ^1^Pre-COVID: before January 31, 2020, when the COVID-19 outbreak was declared as a public health emergency in the U.S. ^2^COVID = January 31, 2020, to May 11, 2023, when the end of the Federal COVID-19 Public Health Emergency was declared in the U.S. N = total number of inspections; n = number in each classification category. Percentages calculated as n/N for year, location, or ET

Comparative analyses of inspection outcomes by location (domestic vs. foreign) and by COVID-19 period (pre-COVID: before January 31, 2020, vs. COVID: January 31, 2020, to May 11, 2023) showed a consistent distribution across classifications. When analyzed by establishment type (CI, Sponsor, CRO, and SI), the inspection outcomes for CIs and Sponsors were similar. In contrast, CROs demonstrated more favorable outcomes, with over 90% of inspections receiving a recommended and final classification of NAI, fewer VAI, and no final OAI (Table 3).

### Comparison of Recommended and Final Classifications of Inspections

Overall, upgrades from recommended NAI or VAI classifications occurred in 2.1% (60/2810), while downgrades from recommended VAI or OAI classifications occurred in 7.8% (39/497) based on the comparison of recommended and final classification of inspections.

Most recommended NAI classifications remained unchanged (97.4%) over the 7-year period, with a small percent upgraded to VAI (2.5%). The percent of upgrades from NAI to VAI remained relatively constant over the 7-year period with a range of 1.7–3.5%. One recommended NAI classification was upgraded to OAI over the 7-year period.

Similarly, the majority of recommended VAI classifications remained unchanged (95.5%), with 4.5% downgraded to NAI. However, there was variation in the distribution of VAI to NAI downgrades by year. Downgrades occurred more frequently in years 2020 (12.2%), 2021 (10.0%), and 2022 (7.3%), compared with the years 2017 to 2019 and 2023 (≤ 2.5%). No recommended VAI classification was upgraded to an OAI.

In contrast, only approximately one third of recommended OAI classifications (8/26, 30.8%) remained unchanged during the 7-year period. The distribution of unchanged recommended OAI classifications varied overtime, ranging from zero in 2017 and 2018 to 100% in 2022 (2/2). Sixteen (16) downgrades were OAI to VAI and 2 were OAI to NAI. The downgrades trended down over the 7-year period with the highest in 2017 (4/4, 100%) and lowest in 2022 (0/2, 0%) (Table  4).

**Table 4:**
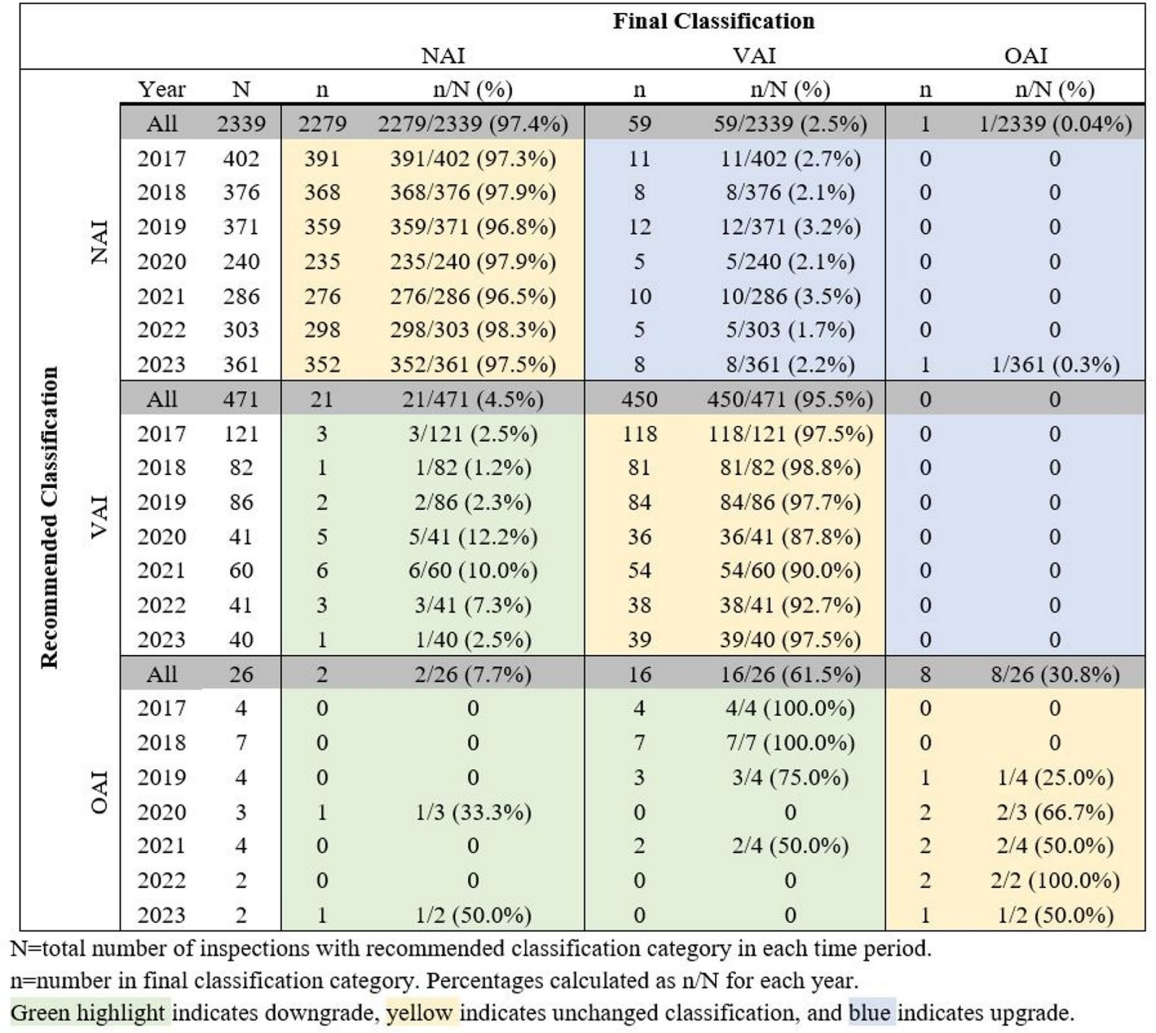
Comparison of Recommended and Final Classifications by Year

### Deficiency Areas for Upgrades and Downgrades

**Fig. 5:**
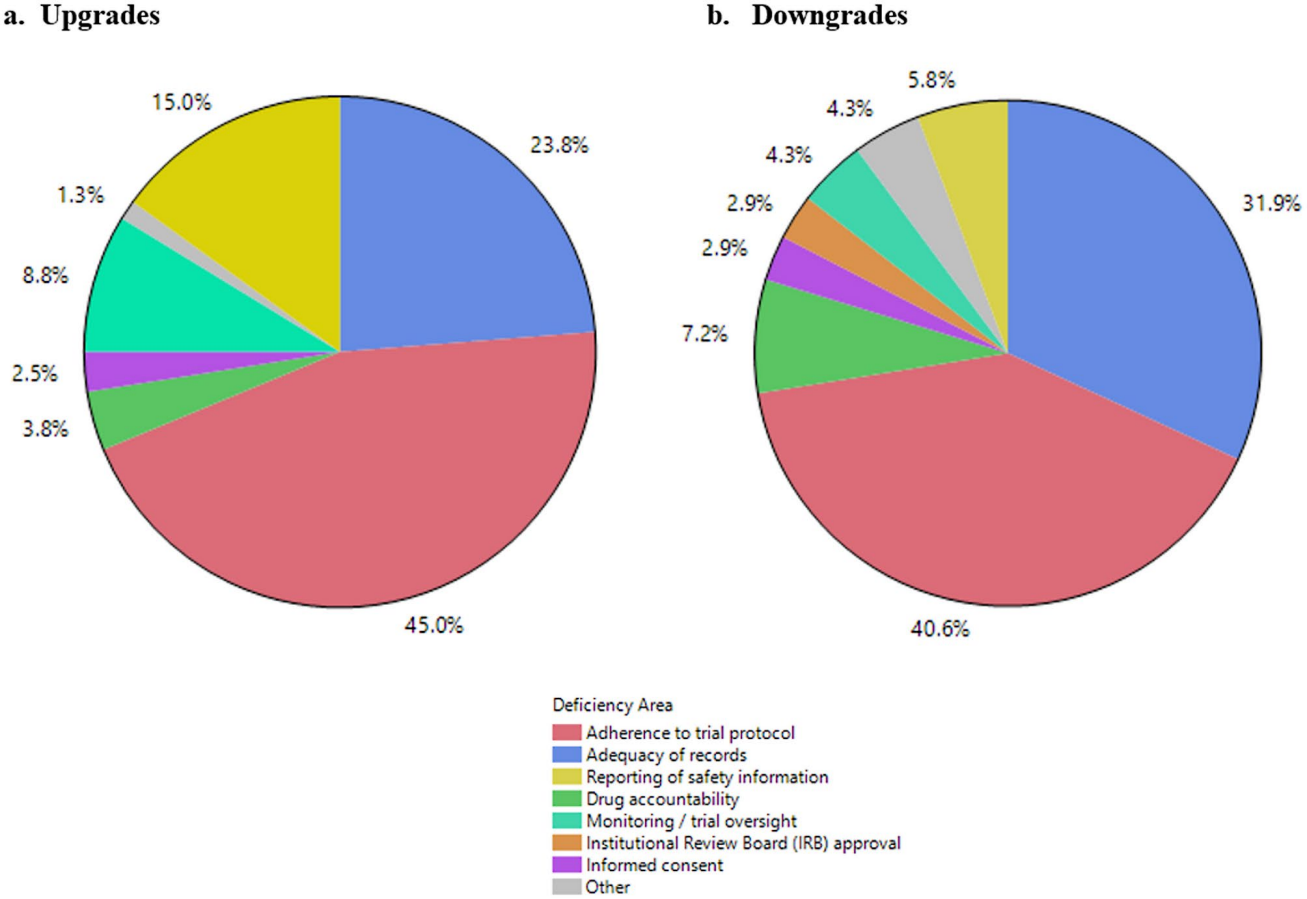
Distribution of Deficiency Area for Upgrades and Downgrades, 2017 to 2023

For the 60 upgraded inspections, the most common deficiency areas were related to adherence to trial protocol (45.0%), adequacy of records (23.8%), and reporting of safety information (15.0%). Deficiency areas related to monitoring / trial adherence, drug accountability, and informed consent each represented less than 9% of the overall final deficiencies (Fig. 5a).

For the 39 downgraded inspections, the most common deficiency areas were related to adherence to trial protocol (40.6%) and adequacy of records (31.9%). Deficiency areas related to drug accountability, reporting of safety information, monitoring/trial oversight, IRB approval, and informed consent each represented less than 8% of the recommended deficiencies (Fig. 5b).

## Discussion

This retrospective study examined the characteristics of review-based routine GCP inspections, the inspection outcomes including whether a Form FDA 483 was issued, the recommended and final classifications, and the related deficiency areas. This analysis focused on the compliance review and classification determinations and not recommendations to the CDER Review Division on the reliability of study data.^2^

The location and type of review-based GCP inspections was fairly consistent from 2017 to 2019, as were the types of applications these inspections supported. After 2019, there was a decline in the number of total and foreign inspections (Fig. 3) as well as a rise in the proportion of inspections supporting applications with a priority review designation (Fig. 4). These trends were likely related to the consequences of the COVID-19 Public Health Emergency declared on January 31, 2020 [12]. FDA prioritized review of COVID-19-related and other mission critical work, including priority review applications providing advances in safety or effectiveness for serious conditions [13–15]. In March 2020, following the President’s declaration of a national emergency, COVID-19 related travel restrictions led to a pause in most foreign and domestic inspections with the exception of mission-critical inspectional work determined on a case-by-case basis (e.g., drugs that were breakthrough therapies, addressed unmet medical needs to treat life-threatening / sight-threatening disease, addressed confirmed drug shortages, medical counter measures, etc.) [16, 17]. Later that year, FDA resumed select domestic inspections utilizing the COVID-19 Advisory Rating System to determine when and where it was safest to conduct prioritized inspections [18]. For example, inspections supporting approval of an application for a high-priority drug product were prioritized as Tier 1 Mission Critical for BIMO inspection [19, 20]. During this time period, FDA also leveraged technologies to conduct RRAs to continue GCP oversight [11]. RRAs were excluded from this analysis; however, the data from RRAs helped support the overall scope of GCP oversight during this time period [10]. Most RRAs were for foreign establishments when inspections were not feasible due to travel restrictions [11], which coincided with the decrease in inspections of foreign establishments.

The percentage of inspections with a Form FDA 483 gradually decreased over the 7-year time period. Reasons for the decline were likely multifactorial. It could reflect an overall shift in GCP compliance at the level of the inspected establishments. For example, advances in technology, including the development and implementation of electronic systems (eSystems) in clinical trial operations (e.g., electronic medical records, electronic case report forms, etc.) and trial oversight (e.g., central and remote monitoring), likely facilitated the ability of regulated establishments to detect and correct errors in real time. While the recent release of multiple FDA guidances reflect the increased use of eSystems [4, 21–25], further research is required to evaluate the connection of these eSystems and the incidence of Form FDA 483s. Other factors which may have resulted in improved GCP compliance during this timeframe include the publication of harmonized GCP Guidelines in 2018 and associated GCP training programs [4, 26].

Analysis of recommended and final classifications noted that the majority of NAI and VAI classifications did not change, with roughly 97% and 96%, respectively, staying the same (Table 4). However, there was a notable increase in VAI to NAI downgrades for the years 2020, 2021, and 2022. This trend in downgrades coincided with the COVID-19 pandemic and could be related to a shift in the FDA’s approach to regulating clinical trial conduct and to inspections during the COVID-19 pandemic. FDA issued guidance in March 2020 to “assist sponsors in assuring the safety of trial participants, maintaining compliance with good clinical practice (GCP), and minimizing risks to trial integrity during the COVID-19 pandemic” [27]. The guidance stated that FDA recognized there may be “unavoidable protocol deviations due to COVID-19 illness and/or COVID-19 control measures” [27]. It recognized that inability to conduct on-site monitoring or delayed monitoring could contribute to protocol deviations and other GCP non-compliance at sites. Finally, it recommended that Sponsors and CROs document such issues and indicated that FDA would consider the unique circumstances of the pandemic control measures in evaluating inspectional observations [27].

The low yearly incidence of recommended OAI and final classifications limit the ability to draw clear conclusions from the observed trends (Table 4). OSI considers not only the scope of regulatory violations but also their significance in the context of the specific trial when determining whether to classify an inspection as OAI. Determining whether regulatory violations exposed participants to an unreasonable or significant risk of illness or injury or compromised the integrity or reliability of critical trial data requires a detailed understanding of the marketing application. Further, the low incidence of OAI classifications reflects that the majority of final OAI classifications are driven by for-cause inspections rather than review-based routine inspections.

Similar areas of inspection deficiencies were reported with inspection classifications that were upgraded or downgraded between 2017 and 2023, with the majority citing nonadherence to the trial protocol or inadequate records (Fig. 5). This likely reflects that these are the most common GCP inspection findings [28]. Changes in final classification related to nonadherence to the protocol may also reflect the nuances in the specific finding, which is highly dependent on the wording of the protocol and its interpretation. Further, OSI’s final classification considers the connection between the specific regulatory violation and risks to trial participants, the impact on the integrity of critical trial data and processes, and the interpretation of safety and effectiveness for the proposed drug product, which may result in upgrades or downgrades. For example, the clinical implications of enrolling a subject that did not meet a protocol-defined inclusion criterion can vary when considered in the clinical context, e.g., exceeding protocol defined maximum subject baseline weight by a small margin without adverse effects versus kidney injury after receiving a trial drug with a narrow therapeutic index in a subject that did not meet minimum baseline weight requirements.

A limitation of this retrospective descriptive study includes lack of data on the total marketing applications (i.e., NDA, BLA, EUA, and supplements with clinical data) submitted by Applicants and reviewed by CDER during the analyzed time period and their breakdown by review type, indication/therapeutic area, etc. Unlike Good Manufacturing Practice (cGMP) inspections, GCP inspections are not requested for all marketing applications. The increased percentage of GCP inspections for priority review applications during the COVID-19 pandemic could be due to an increase in the number of applications submitted to CDER designated as mission critical and priority review, and/or increased requests of GCP inspections for applications with such designation to prioritize FDA’s inspection work [20].

We are encouraged by the analysis of the GCP inspection outcomes for marketing applications. We hope disseminating regulatory and GCP findings such as these may help raise awareness of GCP inspection expectations, and most importantly, strengthen human subject protections and improve clinical trial data quality and integrity.

## Conclusions

FDA conducts an average of 405 (total 2836 in 7 years) review-based routine GCP inspections a year in support of marketing application review in CDER, and the ability to conduct these inspections, particularly outside the U.S., was impacted by the COVID-19 pandemic. FDA prioritized inspections during this time period based on their public health impact and leveraged RRAs [11] to evaluate the reliability and integrity of clinical trial data for marketing applications when inspections were limited. Both inspections and RRAs supported more applications designated as priority review during the pandemic.

Most inspections were final classified as NAI (81.2%) and VAI (18.5%) during the 7-year study period. There was strong agreement between the OII recommended classification and OSI final classification, with roughly 97% of recommended NAI and 96% of recommended VAI classifications receiving the same final classification. There was a consistent overall distribution of classifications when comparing inspections by location and COVID-19 time period. However, there was a notable increase in VAI to NAI downgrades during the pandemic, which may have in part been due to FDA’s consideration of unavoidable protocol deviations due to the impact of COVID-19 on trial conduct.

## Data Availability

Inspection data cannot be shared due to confidentiality agreements.

## Footnotes

1 The Secretary of Health and Human Services issued a determination in 2020 that there was a public health emergency as a result of the COVID-19 pandemic. During this time, FDA authorized unapproved medical products to be used under EUAs which were submitted and reviewed under the associated Investigational New Drug Applications (IND).

2 Regardless of the final inspection classification, OSI makes recommendations to the CDER Review Division on the reliability of clinical trial safety and efficacy data supporting the marketing application based on information collected on GCP inspections.
